# The Distribution and Polymerization Mechanism of Polyfurfuryl Alcohol (PFA) with Lignin in Furfurylated Wood

**DOI:** 10.3390/polym14061071

**Published:** 2022-03-08

**Authors:** Jindi Xu, Dongying Hu, Qi Zheng, Qiulu Meng, Ning Li

**Affiliations:** School of Resources, Environment and Materials, Guangxi University, Nanning 530004, China; kittie7@163.com (J.X.); hdygxu@163.com (D.H.); zhengqist@163.com (Q.Z.); menglib06@163.com (Q.M.)

**Keywords:** distribution, polymerization, polyfurfuryl alcohol, lignin, furfurylated wood

## Abstract

There is increasing interest in furfurylated wood, but the polymerization mechanism between its internal polyfurfuryl alcohol (PFA) and lignin is still uncertain. This paper investigated the distribution of PFA and the feasibility of the polymerization of PFA with lignin in furfurylated balsa wood. The wood first immersed in the furfuryl alcohol (FA) solution followed by in situ polymerization and the distribution of PFA was characterized by Raman, fluorescence microscopy, SEM, and CLSM. Then, the mill wood lignin (MWL) of balsa wood and lignin model molecules were catalytically polymerized with PFA, respectively, studying the mechanism of interaction between PFA and lignin. It was concluded that PFA was mainly deposited in cell corner with high lignin concentration, and additionally partly deposited in wood cell cavity due to high concentration of FA and partial delignification. TGA, FTIR, and NMR analysis showed that the cross-linked network structure generated by the substitution of MWL aromatic ring free position by PFA hydroxymethyl enhanced the thermal stability. New chemical shifts were established between PFA and C_5_/C_6_ of lignin model A and C_2_/C_6_ of model B, respectively. The above results illustrated that lignin-CH_2_-PFA linkage was created between PFA and lignin in the wood cell wall.

## 1. Introduction

Growing wood is net-carbon negative [1] and widely used in construction materials because of its environmentally friendly and technical advantages [2]. However, the major drawback of fast-growing wood is its poor durability [3], which can be effectively improved by impregnation modification. Generally, impregnation with low-molecular-weight resins, such as phenolic resin [4], urea-formaldehyde resin [5], and melamine formaldehyde resin [6], etc., is beneficial to improve the properties of wood, but these resins suffer from the problem of releasing free phenols and free aldehydes. In contrast, furfuryl alcohol is a green modification agent derived from pentose-rich agricultural residues and releases fewer volatile organic compounds or polycyclic aromatic hydrocarbons during the combustion and degradation of FA-modified wood [7,8]. In addition, the distribution of the modifier in the wood and the polymerization with the cell wall material are essential to improve the properties of the impregnated material [9]. These two aspects have been discussed by many scholars.

Numerous studies have shown that the FA monomer can penetrate into the cell wall and that FA resins are more evenly distributed in wood after partial removal of hemicellulose and lignin [10]. Theygesen et al. [11] analyzed by confocal laser scanning microscopy (CLSM) that conjugated PFA was formed in the wood cell wall, which significantly swells the wood cell wall. Li et al. [7] indirectly demonstrated chemically cross-linked FA resin in the wood cell wall by nanoindentation, as both reduced modulus and hardness of the furfurylated wood cell walls were significantly improved. Nevertheless, PFA polymerization with the cell wall in wood is extremely complicated, and it is unclear whether the FA monomer or oligomers are cross-linked with wood cell wall components. Some researchers believe that FA only polymerizes in the cell wall. For example, Yang et al. [12] confirmed that furfuryl alcohol had been polymerized in the wood by NMR, whereas chemical bonds between the FA resins and polymers within cell walls had not formed. Other studies have directly or indirectly demonstrated chemical reactions between lignin and FA monomer or oligomers. Lande et al. [8] have suggested that FA monomers incline to deposit in the cell wall and polymerize in areas with high lignin content, there may be a grafting reaction between lignin and furfuryl alcohol. Ehmcke et al. [13] used cellular ultraviolet micro spectrophotometry (UMSP) images of individual wood cell wall layers to support the hypothesis regarding the coagulation reaction between lignin and FA. The cell walls, especially in the regions with the highest lignin content, showed a significant increase in ultraviolet (UV) absorbance, which indicates a strong polymerization of aromatic compounds. The two studies have shown that the deposition of FA in wood is related to the distribution of lignin in wood, which indirectly indicates the chemical cross-linking of FA and lignin. Furthermore, Nordstierna et al. [14] demonstrated the formation of chemical bonds between cresol, a model lignin, and furfuryl alcohol using NMR. The free vacancies in the benzene ring of lignin generated methylene linkage with FA, which directly proved the chemical combination between lignin and FA. In this study we are illustrating in detail the types of chemical bonding between lignin, FA, and PFA, in addition to testing the previously unknown linkages between PFA and other cell wall polymers, namely cellulose and hemicellulose.

In this study, the balsa wood was first immersed in an FA solution, followed by in situ polymerization to obtain the furfurylated wood. Raman, fluorescence microscopy and SEM were performed to get the distribution of PFA information on different concentration FA and delignification-modified wood. To further discuss the mechanism of interaction between PFA and lignin, the MWL of balsa wood and lignin model molecules were catalytically polymerized with PFA, respectively. TGA, FTIR, and NMR measurements were done to analyze the crosslinking of lignin and FA monomer or PFA. 

## 2. Experimental Sections

### 2.1. Materials

Balsa wood *(Ochroma pyramidale)* was obtained from the market (Guayaquil, Ecuador, South America), the average age of the tree was 6 years, the moisture content was about 10%, and the oven-dried density was 120 kg/m^3^. We took a 20 mm × 20 mm × 20 mm (radial × tangential × longitudinal) sample treated with 1wt% NaClO_2_ solution to obtain delignified wood (DW) [15]; The balsa wood was crushed and ball-milled, then dispersed in dioxane aqueous solution (96:4 volume ratio) to extract the milled wood lignin [16]. 

Furfuryl alcohol (FA), maleic anhydride (MA), 2-methoxy-4-methylphenol, and 2,6-dimethoxy-4-methylphenol were purchased from Shanghai Macklin Biochemical Co., Ltd. (Shanghai, China) Ethyl alcohol (EtOH), Borax (Na_2_B_4_O_7_·10H_2_O), sodium chlorite (NaClO_2_), benzene, dioxane, dichloroethane, ethyl ether, and acetic acid were purchased from Damao Chemical Reagent Factory (Tianjin, China). All chemicals used in this study were as received and without further purification.

### 2.2. Preparation of Furfurylated Wood

The FA impregnation solution was prepared with 2 wt% MA (C_4_H_2_O_3_), 2 wt% Borax (Na_2_B_4_O_7_·10H_2_O), 10 wt% EtOH (C_2_H_5_OH), (10, 20, 30, 40, and 50 wt%) FA (C_5_H_6_O_2_), and DI water. Prior to the impregnation, the wood samples were divided into native wood (CW) and delignified wood (DW), with 10 samples in each group. All samples were oven-dried, and the mass was recorded as m_0_. As-prepared solutions were impregnated into the wood samples under vacuum at –0.1 MPa for 24 h and then in the atmosphere for 48 h. After impregnation, the samples were wiped with tissue paper to remove excess solutions on the surface. To avoid solution evaporation during the curing stage, the samples were wrapped in aluminum foil, then cured in an oven at 103 °C for 3 h, allowing full polymerization of FA within the wood matrix. Afterward, the aluminum foil was removed. The samples were heated from 40 to 103 °C at a rate of 10 °C/h until oven-dry (m_1_) to obtain furfurylated wood (CFW) and furfurylated delignified wood (DFW). The weight percentage gain (WPG) of furfurylated wood was calculated accordingly,

(1) $$\mathrm{WPG}=\frac{m_{1}{\;-\;m}_{0}}{m_{0}}\;\times \;100\%$$

### 2.3. Preparation of Furfurylated Lignin

MWL and FA were evenly mixed at 1:6 (wt%:wt%) and solidified after 24 h of reaction at 103 °C to obtain the PFA–lignin complex, LPFA, and the chemical bond between wood and furfuryl alcohol was investigated by an NMR test.

Two model molecules, 2-methoxy-4-methylphenol (A) and 2,6-dimethoxy-4-methylphenol (B), were homogeneously mixed 1:1 (wt%:wt%) with FA solution and completely cured after 24 h of reaction at 103 °C to derive PFA–lignin model molecule composites A-LPFA and B-LPFA, respectively, for further analysis of lignin and PFA chemical bonding.

### 2.4. Characterization

A Raman microscope (Renishaw, London, UK) equipped with a 50× microscope objective and a linear-polarized 633 nm laser was used to examine the distribution of lignin. The wavenumber range of 3000–2000 cm^−1^ at a resolution of 2 cm^−1^. For mapping, 1 μm steps were chosen, and every pixel corresponded to one scan. 

The morphology of the delignified wood and furfurylated wood was characterized by Sigma 300 field emission gun scanning electron microscope (ZEISS, Oberkochen, Germany). 

The microscopic distribution of PFA resin within the furfurylated wood was examined using a Image Z2 fluorescent microscope (ZEISS, Oberkochen, Germany) with a 40× microscope objective. The small pieces of wood from a specimen with size of 3 mm × 5 mm × 0.2 mm were stained with 0.5% toluidine blue solution for 10 min to suppress the autofluorescence of lignin for acquiring images. 

The microscopic distribution of PFA resin within the furfurylated wood was examined using LEICA-TCS-SP8MP confocal laser scanning microscopy (CLSM) (Leica, Wetzlar, Germany) with a 63× (oil immersion) objective lens. The excitation laser wavelength was 633 nm, and the detector range was 650–700 nm.

The thermal degradation behaviors of MWL, LPFA, and PFA were investigated using a DTG-60 (H) thermogravimetric analyzer (Shimadzu, Kyoto, Japan) from room temperature (25 °C) to 800 °C at a heating rate of 10 °C/min in a flowing nitrogen atmosphere of 100 mL/min. 

FTIR were recorded on a Thermo Scientifific Nicolet iS50 spectrophotometer (Waltham, MA, USA) over the wavenumber range of 4000–400 cm^−1^ at a resolution of 4 cm^−1^, using the KBr pellet method. 

The polymerization mechanism of PFA resin, LPFA, A-LPFA, and B-LPFA were investigated by ^13^C NMR analysis. The Spectra were recorded on a Avance HD500 spectrometer (Bruker, Karlsruhe, Germany), and the DMSO-d6 cross-peak at δC13 39.52 ppm was used as an internal reference. Solid-state NMR spectra were measured at room temperature with an Bruker III 400 NMR spectrometer (Bruker, Karlsruhe, Germany) fitted with a 4 mm magic-angle spinning (MAS) probe head. 

## 3. Results and Discussion

### 3.1. Distribution of PFA Resin in Wood

**Figure 1:**
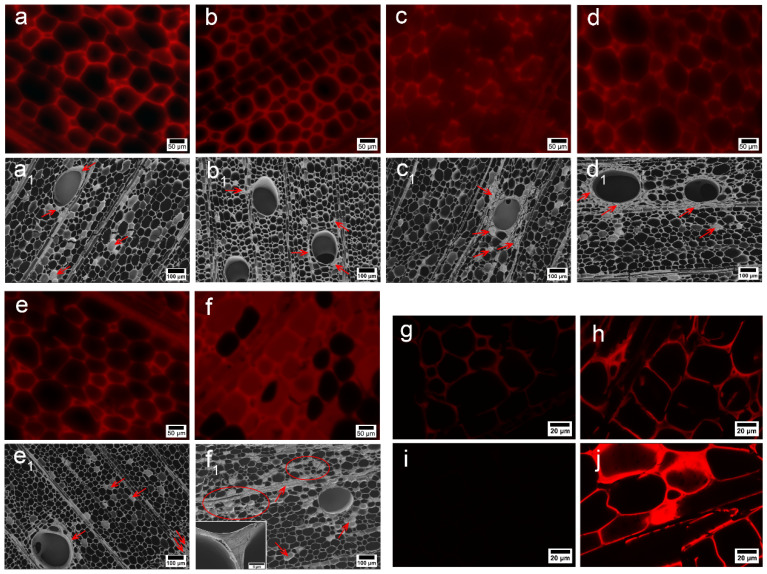
Surface fluorescence microscopy of 10% FA CFW (**a**), 20% FA CFW (**b**), 30% FA CFW (**c**), 40% FA CFW (**d**), 50% FA CFW (**e**), and 50% FA DFW (**f**); SEM image of 10% FA CFW (**a_1_**), 20% FA CFW (**b_1_**), 30% FA CFW (**c_1_**), 40% FA CFW (**d_1_**), 50% FA CFW (**e_1_**), and 50% FA DFW (**f_1_**); CLSM image of CW (**g**), CFW (**h**), DW (**i**), and DFW (**j**).

**Figure 2:**
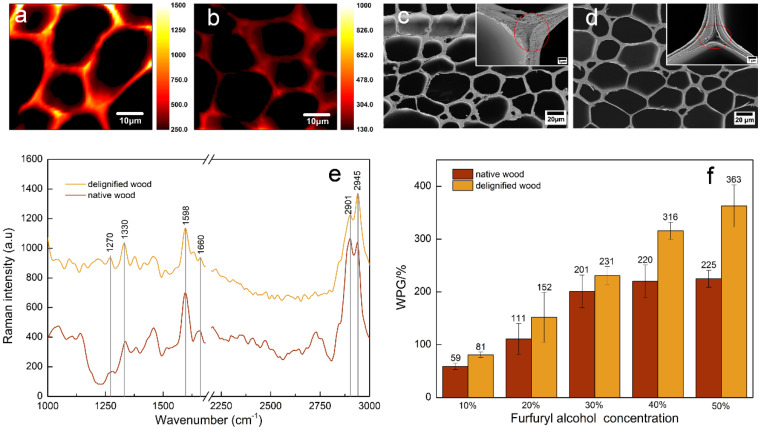
(**a**,**b**)—Raman images of lignin distribution of CW and DW, respectively; (**c**,**d**)—SEM analysis of CW and DW, respectively; (**e**)—average Raman spectra extracted from the cell walls of CW and DW; (**f**)—WPG for different concentrations of furfuryl alcohol in CW and DW.

In order to study the distribution of PFA resin in wood, various characterization methods were used. Firstly, fluorescence imaging of furfurylated wood was conducted under a fluorescence microscope (Figure 1). To effectively suppress the autofluorescence of lignin in cell walls, staining treatment with toluidine blue dye was performed in advance. The bright red spots in the figure are FA resin filled in the cell wall of the wood, and the fluorescence intensity reflects the filling degree of FA resin. The increase in FA concentration improved the opportunity for PFA resin to adhere to the interior of the wood, and more PFA resin polymerized on the cross section of the wood, contributing to a WPG of 225% for the 50% FA CFW (Figure 2f). In addition, fluorescence intensity analysis performed on the fluorescence image by Image J (Table 1) showed that the penetration area of 50% FA CFW was 110,196.67 μm^2^, accounting for 35.19%. The infiltration area increased by 58.58% compared to that of 10% FA CFW. The SEM micrographs shown in Figure 1 exhibit the distribution of PFA resin. The figure reveals that in the low concentration of CFW, only a small amount of the lumen was filled with PFA resin, and most of it may have been present in the cell wall. As the concentration increased, the FA resin inclined to deposit near the vessels and rays, as shown by the arrows in Figure 1. On the one hand, the vessels are conduits for the delivery of water and nutrients to the wood, pits in the inner wall of the vessels are connected to the radial tracheids, allowing FA to penetrate radially and deposit in the cell cavity [17]. On the other hand, FA monomers easily volatilize during heating and curing, causing fewer deposits of PFA resin in the vessels and cell cavity. Finally, CLSM fluorescence imaging was performed on the specimens in order to have a clearer observation of the distribution of PFA resin in the wood microstructure. As shown in Figure 1g, there was a faint fluorescence on the cell wall of CW, which was the autofluorescence of lignin, and the fluorescence disappeared after delignification treatment. In contrast, CFW fluoresced strongly in the cell wall and part of the cell cavity. This suggests that the PFA resin was not only deposited in the cell lumen, but may have also been present in the cell wall region.

According to previous reports, FA monomers tend to be deposited on the cell walls in regions with high lignin content. To that end, in this study we partially delignified balsa wood to investigate whether there was a correlation between the distribution of lignin and PFA in wood. In the Raman spectrum of CW and DW (Figure 2e), the bands belonging to lignin structures were annotated at 1270, 1330, 1598, 1660 and 2945 cm^−1^ [18]. The two band at 1270 and 1330 cm^−1^ were attributable to G and S units in balsa samples, where the G units were assigned to aromatic ethers stretch, and S units were attributed to bending vibration of phenolic hydroxyl lignin [19], respectively. Other lignin features were detected at 1598, 1600, and 2945 cm^−1^. There was a strong band at 2945 cm^−1^ from the C–H stretch of the methoxyl groups of the lignin. The peak around 1598 cm^−1^ pointed to the lignin stretching vibrations of the aromatic ring, and that at 1660 cm^−1^—to ring-conjugated C=C bonds in coniferyl alcohol units [20,21]. It follows that the characteristic peak absorption intensity of lignin decreased with delignification treatment. The distribution of lignin can be visualized in Figure 2a,b by integral calculation in the range of 1540–1700 cm^−1^. Raman imaging showed that high fluorescence intensity of the cell corner (CC) and compound middle lamella (CML) of fibers in untreated samples. Chlorine dioxide generated by acidic sodium chlorite oxidizes phenolic lignin to o-quinone and p-quinone structures [22,23] while cutting the lignin and cellulose, hemicellulose into water-soluble small molecules, thus removing lignin and dropping the Raman intensity, which is more reactive CML than in CC. It can be seen that the CC was highly lignified, and lignin was difficult to remove.

DW was impregnated with PFA resin and observed by a fluorescence microscope and CLSM. It was found that the fluorescence intensity at CC in the fiber cells without PFA in the cell cavity was stronger, suggesting a possible correlation between PFA and lignin. Moreover, a large amount of FA resin filled the cell cavity of DFW compared to that of CFW (Figure 1f), resulting in a WPG of 363% for DFW with 50% FA, which was 61% higher compared to that of CFW, due to formation of a nanonetwork structure after acidic sodium chlorite treatment, exposing more active sites and increasing the permeability of the cell wall [24]. Previous studies have also attested that delignification promotes the impregnation of the modifier and its interaction with cellulose [25]. The analysis of fluorescence intensity calculation also indicated that the delignification treatment significantly increased the glue spots on the DFW cross section. The permeable area of 50% FA DWF was 210,576.16 μm^2^, accounting for 67.24%, which increased by 91.08% compared to that of 50% FA CFW. In addition, SEM images displayed a macroscopic shape, and the microscopic honeycomb-like cell wall architecture of native wood was preserved in delignified samples (Figure 2d). The DW cell wall changed into thin, and deformation came into being. After lignin removal, there was a phenomenon where cracks presented in the cell corner, and the cell wall was stratified (Figure 2d). However, these cracks were filled with PFA resin after impregnation with FA (Figure 1f_1_). Moreover, PFA resin was also found in the intercellular layer, and the cell walls of furfurylated wood were thickened. 

### 3.2. The Polymerization Mechanism of PFA and CW

**Figure 3:**
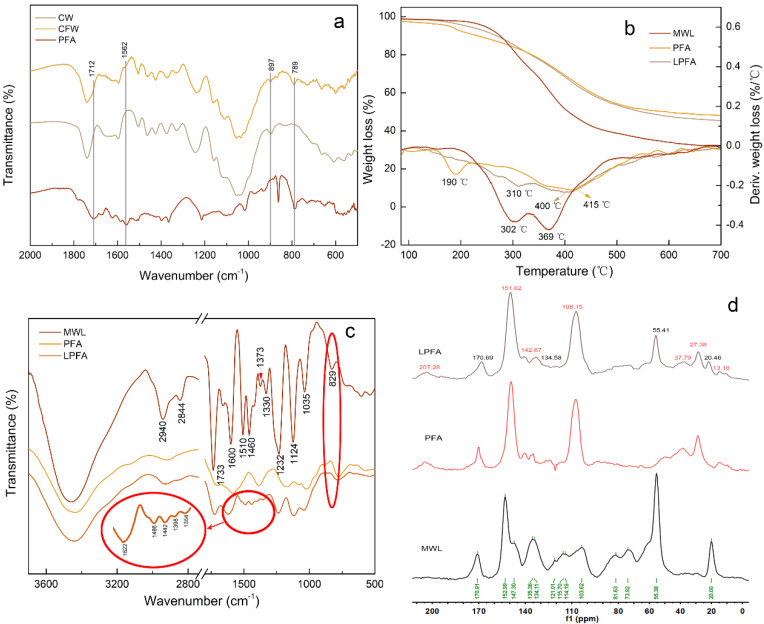
(**a**)—FTIR spectra of CW, CFW, and PFA; (**b**)—TGA and DGA of MWL, PFA and LPFA; (**c**)—FTIR spectra of MWL, PFA and LPFA; (**d**)—^13^C NMR spectra of MWL, LPFA, and PFA.

In order to investigate the possible chemical functional groups changes and chemical reactions between PFA and lignin, FTIR spectra of CW, CFW, and PFA were used to obtain more information (Figure 3a). There were new bands at 1562 and 789 cm^−1^ in CFW compared to those of CW, assigned to skeletal vibration of 2,5-disubstituted furan rings and conjugated C=C species [8], respectively. There was also a little peak at 1712 cm^−1^ of furfurylated wood attributed to the C=O stretch of γ-diketones formed from hydrolytic ring opening of the furan rings, covered up by 1741 cm^−1^ assigned to C=O stretching vibrations in unconjugated ketones, carbonyls, and esters. In addition, the intensity of the small peak at 897 cm^−1^ assigned to the β-glycosidic linkages between the sugar units decreased compared with that of control wood, suggesting that acid hydrolysis of hemicelluloses occurred during the furfurylation of wood [20]. This demonstrated that the polymerization of FA was successfully completed within wood, but there was insufficient evidence of chemical reactions between PFA and wood molecules. Based on previous reports and the distribution of PFA resin in wood in this paper, it is known that there may be a relationship between PFA and lignin. Therefore, MWL and lignin model molecules were used for catalytic polymerization with PFA, respectively, to further explore the polymerization between PFA and lignin.

### 3.3. The Polymerization Mechanism of PFA and MWL

Firstly, the thermal degradation stability of MWL, PFA, and LPFA was obtained by thermogravimetric analysis. The thermograms (TGA) and derivatives of thermograms (DTA) are gathered in Figure 3b. The DTA curves showed that the main peak around 415 °C for PFA was attributed to the degradation of methylene and the furan ring in the PFA chain; the peak at 190 °C was owing to the volatiles that evolved during further condensation polymerization of the PFA resin [26]. MWL lost a little weight before 200 °C, mainly water molecules and some small molecule impurities in the sample, and its decomposition temperature range was mainly between 200 and 500 °C. The weight loss was faster at 302 and 369 °C, and the final carbon residue was 31%. LPFA started to decompose slowly at about 120 °C, and the decomposition rate was faster at about 310 and 400 °C. The carbon residue rate was 44%, which was 42% higher than that of MWL, with a slower degradation rate and higher thermal oxidation degradation temperature. This denoted that PFA with a relatively stable furan ring structure polymerized with MWL to form a more complex cross-linked network structure, resulting in better thermal stability of LPFA.

Next, FTIR was used to qualitatively analyze the functional groups on the structure of MWL, PFA, and LPFA, and the spectra are shown in Figure 3c. The characteristic absorption of each functional group in MWL was mainly concentrated in the range of 3800–800 cm^−1^ [27], with typical infrared spectral characteristics of lignin, that is, benzene C=C skeleton vibration occurred at 1600 and 1510 cm^−1^. The absorption at 2940 and 2844 cm^−1^ arose from the C-H stretching vibrations in the methoxyl group and methyl or methylene groups, respectively [28]. The strong band at 1232 cm^−1^ was indicative of the absorption peak of p-hydroxyphenyl. The band at 1035 cm^−1^ originated from the C-O stretching vibration in primary alcohol and C-H vibration on G unit, and 1733 cm^−1^ due to C=O stretching vibration in unconjugated ketones and carboxyl groups. Additionally, the sharp absorption band at 1124 cm^−1^ and the absorption bands at 829 and 1330 cm^−1^ in these spectra indicated that balsa MWL had typical wood quality characteristics of hardwood [29]. Obviously, the spectra of LPFA were offset between 1600 and 1300 cm^−1^, such as the C=C vibration at the benzene ring and the characteristic peak shift of the S unit aromatic ring, marked in Figure 3c. Moreover, the O-H stretching vibration of phenolic hydroxyl groups was also affected. Consequently, condensation with PFA hydroxyl may have existed at the free position on the aromatic ring of MWL, leading the peak position of the aromatic ring being affected. Furthermore, the absorption of the Syringyl unit C-H (829 cm^−1^) disappeared in LPFA, indicating that the C_2_/C_6_ position of the S unit was occupied by the other groups. In summary, the MWL of Balsa was successfully extracted with a large number of Syringyl units and a small amount of Guaiacyl units, and the hydroxyl group of PFA and the free position of MWL aromatic ring were condensed.

**Figure 4:**
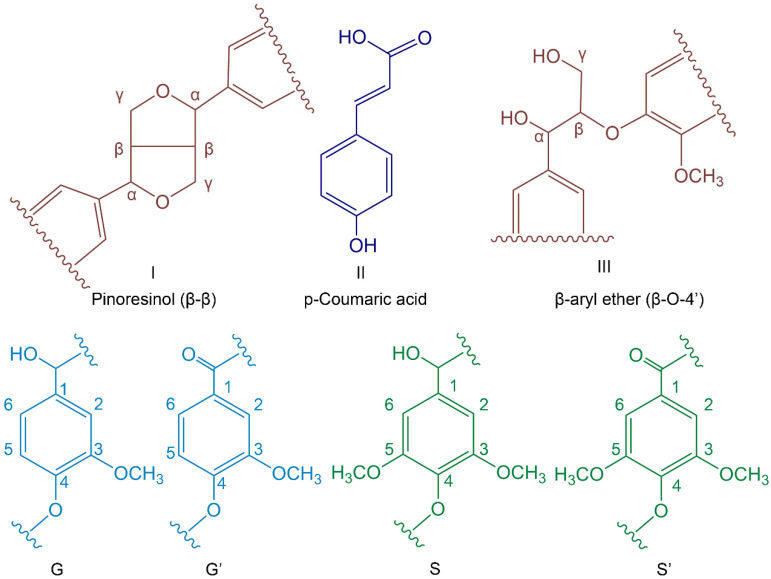
Main structures present in balsa wood MWL.

The structures were further examined, and ^13^C NMR was utilized to characterize MWL and LPFA, as shown in Figure 3d. The attribution of related signals refers to Holtman [30] and Pang [31], as shown in Table 2, and the main unit structures of MWL are shown in Figure 4. The intensities of the signal belonging to methyl (δ20 ppm) in the aliphatic region (10–50 ppm) were significantly high. In the side chain region (50–90 ppm), the intensities of the methoxy group (δ55.38 ppm) presented prominently, and the signals of C_α_ (δ73.92 ppm) and C_β_ (δ81.63 ppm) of β-O-4′ aryl ether linkages (III), which are typical bonds of natural lignin, were obvious. In the aromatic region (100–160 ppm), Syringyl (S) and Guaiacyl (G) lignin units, main characteristic units of hardwood lignin, could be detected. The S unit presented a C_2_/C_6_ (δ103.62 ppm) chemical shift signals as well as a strong signal of C_3_/C_5_ (δ152.99 ppm). The chemical shift signals of C_1_ (δ135.36 ppm) were strong, and C_3_, C_5_, and C_6_ were weak on the G unit. In general, the S unit signal was more intense than that of the G unit, which was in line with the results of the FTIR spectrum. Comparative analysis of LPFA NMR spectra of MWL after furfurylation suggested that it had more PFA polymer signals, and the attribution of the relevant signals is given in Table 3. The signals at δ151.62 and δ108.15 ppm in the PFA polymer were stronger than MWL signals, representing C_1_ and C_3_ on the furan ring in the PFA chained structure, respectively. The chemical shifts of -CH_2_- (δ27.38 ppm) between the furan rings were also remarkable. The chemical shift signals of the methoxy group (δ55.41 ppm) and p-coumaric acid (δ170.69 ppm) were also obtained in LPFA NMR spectra, along with a small amount of methyl (δ20.46 ppm). In brief, no new chemical shift signals were found in the LPFA NMR, except for those of PFA and MWL. The possible reasons were as follows: Firstly, it is proposed that the C_5_ position of the G unit was more likely to attract the attack of carbon atoms on FA and participate in electrophilic aromatic substitution reactions [14,32], while the MWL of Balsa was dominated by the Syringyl unit, and the content of Guaiacyl unit was less, thus, no cross-linking reaction between Balsa lignin and PFA was detected. Secondly, no new chemical shift signal was found due to the complex structure unit of lignin as well as the low resolution of solid-state NMR resulting in broad peak positions, leading to overwriting of new chemical shifts generated by the PFA and MWL reactions. 

### 3.4. The Polymerization Mechanism of PFA and Lignin Model Molecules

**Figure 5:**
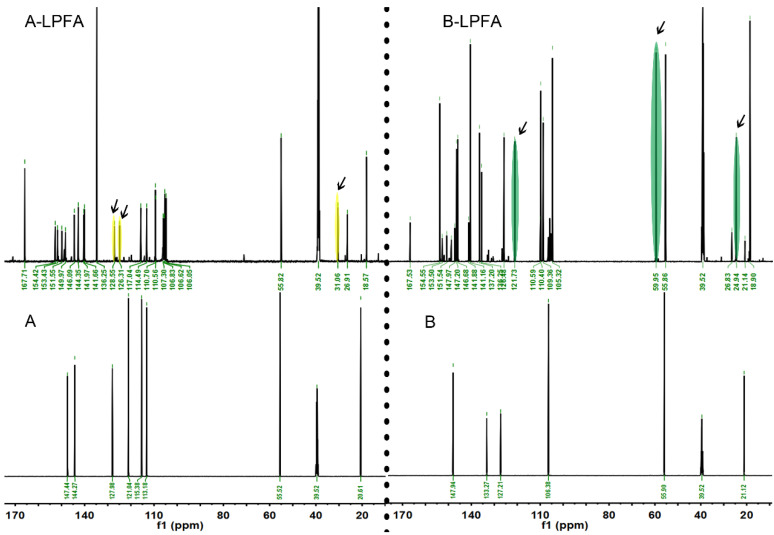
^13^C NMR spectra of lignin model (**A**,**B**) and (**A-LPFA**,**B-LPFA**).

**Figure 6:**
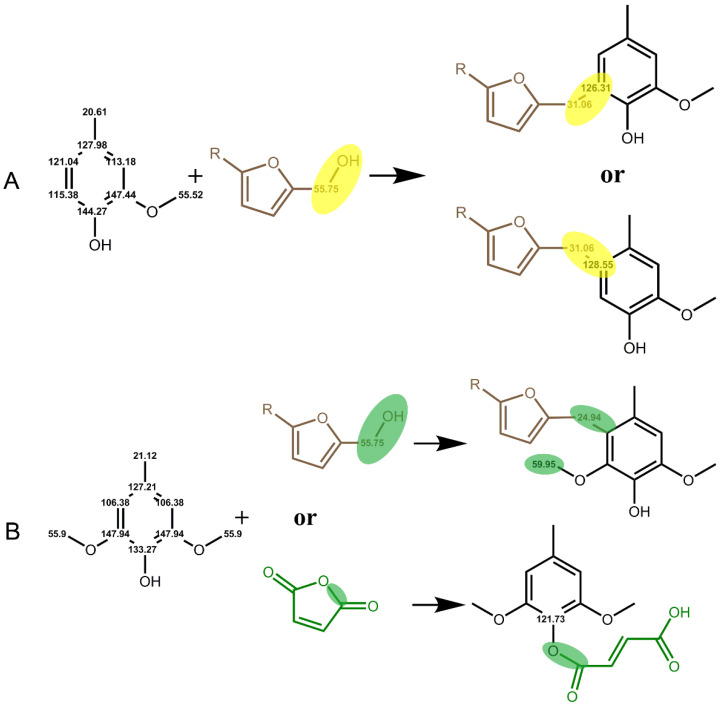
Hypothesized reaction between FA and lignin models (**A**,**B**). Substituent R is either a poly(furfuryl alcohol) chain or a hydrogen.

The main MWL unit of Balsa wood is the S unit, and the structure of pure lignin is too complex, resulting in an obscure mechanism of MWL and PFA. Therefore, the mechanism of interaction with PFA was explored by simplifying lignin, for example, by using a lignin model molecule. In this paper, two lignin major unit model molecules, 2-methoxy-4-methylphenol (A) and 2,6-dimethoxy-4-methylphenol (B), were mixed with an FA impregnating solution at 1:1 (wt%:wt%) and thoroughly reacted at 103 °C for 24 h to obtain the furfurylated lignin models A-LPFA and B-LPFA, respectively. A-LPFA and B-LPFA were dissolved in DMSO-D_6_, ^13^C NMR detection (Figure 5) was performed, and the chemical structure changes in the furfurylated model were analyzed (Figure 6).

Model A was catalytically polymerized with FA to obtain A-LPFA. In addition to chemical shift signals of model A and PFA, a new polymer ^13^C signal was detected in modified model A (Figure 5A-LPFA), which was not observed for any other combinations or treatments of the starting materials. Significantly, excluding the -CH_2_- links (δ26.91 ppm) between the furan rings, one of the new methylene signals (δ31.06 ppm) appeared as indicated by the arrow, which belonged to a covalent bridge between model A and PFA resin. Based on the NMR spectra, it was speculated that the covalent bridges between model A and PFA resin might be at the position of C_5_ and C_6_ of model A (Figure 6). As an electrophilic aromatic substitution reaction, both the hydroxyl group and the methoxy group on the benzene ring of model A could be utilized as the positioning group. According to the density functional theory (DFT) model calculated by Barsberg [33] et al., the enthalpy of C_5_ and C_6_ positions of cresols in ethanol and aqueous solution do not differ appreciably, indicating that both positions on the benzene ring may be connected to PFA. Moreover, the NMR spectra showed the chemical shifts of C_5_ (δ126.31ppm) and C6 (δ128.55 ppm) shifted toward lower magnetic by the PFA connection. Nordstierna [14] et al. also indicated that -CH_2_-, as a link between cresol and FA, can be observed in the chemical shift signal at δ31.7 ppm.

Model B was catalytically polymerized with FA to obtain B-LPFA, and a new polymer ^13^C signal were detected (Figure 5B-LPFA), one for new methylene signals (δ24.94 ppm) appeared as indicated by the arrow, which belonged to the covalent bridge between model B and PFA resin. The second was δ121.73 ppm, which was inferred to be the chemical shift of an esterification reaction of maleic anhydride with the hydroxyl group on the benzene ring, and not FA (Figure 6). Guigo [34] et al. suggested that the connection of FA with the hydroxyl group on C_4_ produces a new chemical shift around δ66 ppm, but no such signal was detected in this experiment. Therefore, it can be inferred that the entry of maleic anhydride shifts the C_4_ chemical shift on the benzene ring of model B to the high magnetic field. Another new chemical shift signal was δ59.95 ppm, which was influenced by the electrophilic substitution reaction between FA and model B at C_2_/C_6_, causing the methoxyl carbon on model B move to a lower magnetic field. According to the directing effect and reactivity principle of electrophilic substitution reaction on the aromatic ring, the activity of the hydroxyl group was higher than that of the methoxyl group, but since the para position of hydroxyl group was occupied, the methoxyl group acted as the directing group and developed -CH_2_- bridging with PFA at its adjacent and para positions. The results of FTIR spectra also testified that the disappearance of C-H absorption of the Syringyl unit was associated with the access of PFA hydroxymethyl. It is thus proposed that the hydroxymethyl group of PFA had a new chemical bond after condensation at the free position of the aromatic ring of model B, and maleic anhydride catalyst may have also reacted with model B.

In conclusion, model A and B, representing the Guaiac unit (G) and the Syringyl unit (S), respectively, could basically explain the formation of lignin in the cell wall, both of which could have an electrophilic aromatic substitution reaction with PFA, indicating the presence of lignin-CH_2_-PFA and the chemical cross-linking between PFA and wood cell wall polymers.

## 4. Conclusions

In this work, a variety of characterization methods were utilized to display the distribution of PFA in furfurylated wood, and a new chemical link was established between PFA and lignin, demonstrated by the reaction of two types of lignin with it. Raman imaging, fluorescence microscopy, SEM, and CLSM observations revealed that PFA presented more in the intercellular layer and the cell corner where the concentration of lignin was higher than that in the cell cavity. In addition, we found that delignified wood allowed for better infiltration by PFA compared to that in the control, but the mechanism of action was not investigated, and we will continue to study it in the future. MWL and lignin model molecules were catalytically polymerized with PFA, respectively, and TGA and FTIR measurements showed that the cross-linked network structure generated by the substitution of the MWL aromatic ring-free position by PFA hydroxymethyl enhanced the thermal stability. NMR analysis confirmed that there was no obvious connection signal between FA and MWL in LPFA, nevertheless, both methoxy and hydroxyl groups on the lignin model molecules could be as directing groups to activate the benzene ring and attract carbocation on PFA to attack, and -CH_2_- covalent connection with PFA came into being. In addition, the catalyst maleic anhydride may have also esterified with the phenolic hydroxyl group.

## Figures and Tables

**Table 1:** Distribution of PFA at different concentrations in the cross section of wood.

Samples	Infiltration Area (μm^2^)	Ratio of Permeable Area (%)
CW	4.10	6.07
dyed CW	2.96	4.39
10% FA CFW	69,484.04	22.19
20% FA CFW	84,619.40	27.02
30% FA CFW	93,675.93	29.91
40% FA CFW	106,763.16	34.09
50% FA CFW	110,196.67	35.19
50% FA DFW	210,576.16	67.24

CW was native wood, CFW and DFW were FA modified native wood and delignified wood, respectively.

**Table 2:** Assignment of the ^13^C CP/MAS NMR spectra of MWL.

Peak	δ	Assignment	Peak	δ	Assignment
1	170.91	C=O in_α_	8	114.19	C_3_/C_5_ in G’
2	152.99	C_3_/C_5_ in S	9	103.62	C_2_/C_6_ in S
3	147.30	C_3_/C_5_ in S’	10	81.63	C_β_ in III, C_α_ in I
4	135.36	C_1_ in G	11	73.92	C_α_ in III
5	134.11	C_1_ in G’	12	55.38	-OCH_3_
6	121.01	C_6_ in G	13	20.00	-CH_3_
7	115.70	C_3_/C_5_ in G	——	——	——

**Table 3:** Assignment of the ^13^C CP/MAS NMR spectra of LPFA.

Peak	δ	Assignment
1	207.28	C=O in Furan ring opening
2	170.69	C=O in p-Coumaric acid
3	151.62	C_1_ in chain structure of PFA, C_3_/C_5_ in S
4	142.67	C_4_ in chain structure of PFA
5	134.58	C_1_ in G/G’
6	108.15	C_3_ in chain structure of PFA
7	55.41	-OCH_3_ in MWL
8	37.79	-CH_2_ in reticular conformation of PFA
9	27.38	-CH_2_ in chain structure of PFA
10	20.46	-CH_3_ in MWL
11	13.18	-CH_3_ in chain structure of PFA

## Data Availability

The data presented in this study are available on request from the corresponding author.
